# Validation of the Italian version of the Neuroception of Psychological Safety Scale (NPSS)

**DOI:** 10.1016/j.heliyon.2024.e27625

**Published:** 2024-03-16

**Authors:** Andrea Poli, Mario Miccoli

**Affiliations:** Department of Clinical and Experimental Medicine, University of Pisa, 56126, Pisa, Italy

**Keywords:** Psychological safety, Polyvagal theory, Neuroception, Body perception, Psychological trauma, Psychometrics

## Abstract

Research on the neuroscience of fear in both humans and non-humans has suggested that a lack of acquisition of safety cues might be a biological hallmark of posttraumatic stress disorder (PTSD). Danger perception, and in particular, feeling as one's own life is in danger, is thought to represent a major predictor of PTSD. Persistent danger perception is concurrently associated with a persistence of lack of safety. However, despite several research efforts, no validated psychometric tools exist regarding psychological safety as a unique core construct in the domain of a soothing-contentment system. By including social, compassionate, and bodily components, the Neuroception of Psychological Safety Scale (NPSS), neurophysiologically rooted in the polyvagal theory, aims to specifically assess psychological safety. Originally developed in English, we employed a rather large non clinical sample to validate our Italian translation of the NPSS (n = 338) and the scale was found to retain a three-factor structure. In light of its positive moderate correlations with the Unconditional Self-Kindness Scale (ρ = 0.376) and the Self-Compassion Scale-Short-Form (ρ = 0.481), good convergent validity and robust psychometric properties were shown by the NPSS. The Subjective Traumatic Outlook Questionnaire (ρ = −0.283) and the three subscales of the Body Perception Questionnaire-22—Body Awareness (ρ = −0.103), Supradiaphragmatic Reactivity (ρ = −0.234), and Body Awareness/Subdiaphragmatic Reactivity (ρ = −0.146)—were found to have weak negative correlations with the NPSS, which further demonstrated its good discriminant validity. Eventually, the NPSS was found to show good test-retest reliability (intraclass correlation coefficient = 0.922; three-week time interval), and its usage is fostered in clinical and research contexts where the evaluation of psychological safety is of relevance.

## Highlights

• We validated the Italian version of the Neuroception of Psychological Safety Scale (NPSS).
• Exploratory Structural Equation Modeling suggested a three-factor model solution.
• Confirmatory Factor Analysis revealed an adequate fit for the three-factor model.
• Convergent and discriminant analyses supported the construct validity of the NPSS.
• Test-retest, intraclass and split-half analyses supported the reliability of the NPSS.

## 1 Introduction

Danger perception and, in particular, perceiving one's own life threatened, is believed to represent a major predictor of posttraumatic stress disorder (PTSD) after experiencing a traumatic wound [1], as well as a maintenance factor [2], and persistent danger perception may lead to hypervigilance. Accordingly, compared to controls selected for the absence of a well-documented history of psychological trauma [[3], [4], [5], [6]] or emotional disturbances reported by their educators or parents, children and adolescents with PTSD differentially directed processing resources away from depression-related stimuli and toward socially threatening stimuli [7]. Persistent danger perception is concurrently associated with a persistence of lack of safety, and research on the neuroscience of fear in both humans and non-humans has suggested that a lack of acquisition of safety cues might be a biological hallmark of PTSD [[8], [9], [10], [11]].

The Veterans Health Administration and Department of Defense, and the American Psychological Association released treatment guidelines for PTSD in 2017 that include a list of suggestions for healthcare professionals who are involved in the treatment of PTSD patients. Prolonged Exposure, Cognitive Processing Therapy, and trauma-focused Cognitive Behavioral Therapy (CBT) were all highly recommended. Each of these therapies has a strong body of research and they are trauma-focused, which means that these therapies deal with the traumatic event's memories or associated thoughts and emotions [[12], [13], [14]]. However, none of these therapies directly address the issue of safety feelings. Safety issues for PTSD were addressed by a specific form of CBT, named “Seeking Safety” [15]: the term referred to the fundamental principle of the therapy, which stated that in order to recover from PTSD safety had to be given priority. Safety was described as the creation of a support network and self-defense against risks related to the disease (e.g., domestic violence). Themes that were deemed to be most important for this patients were addressed in all sessions: for instance, seeking support, taking care of oneself, establishing boundaries [15]. Results of “Seeking Safety” treatment showed significant improvement for women and adolescent girls with PTSD and substance use disorder (SUD) [16,17] and in veterans with PTSD and SUD [18,19].

In healthcare work environments, the issues of psychological safety has been taken into account defining it as the possibility for workers to express themselves in a professional setting without worrying about shame or judgment from others [20]. This research line highlights that personal, team-based, or organizational elements may all affect psychological safety. While most of the prior research has been on the leaders' role in fostering psychological safety, a safe environment may be produced by any team member [20]. In addition, among emergency nurses, psychological safety has been found to represent a main factor potentially affecting their patient safety competency [21]. Accordingly, a composite measure, combining the advantages of observational and survey measurements, has been developed designed for use by healthcare teams [22]. Overall, a conceptual analysis regarding 88 articles investigating psychological safety in healthcare settings was able out to point out five factors critically contributing to psychological safety: solid interpersonal bonds, perception of the effects of interpersonal risk-taking, group-level phenomena, a safe work atmosphere where taking interpersonal risks is encouraged, and a non-punitive culture [23].

As highlighted by Sweeney et al. [24], organizational and mental health services need a paradigm shift from trauma-uninformed- to trauma-informed mental healthcare approaches in order to avoid re-traumatization of patients by trauma-uninformed staff, and to avoid the vicarious traumatization of staff by the patients' traumatic expression and by practices like confinement and isolation. In fact, the emergence of trauma-informed techniques and therapies may be partially related to a plethora of studies indicating the pervasiveness of trauma in society, its strong correlation with mental health, and its high cost as a public health concern. When therapeutic help is correctly offered using a trauma-informed technique or therapy, the primary change is to ask yourself, ‘What happened to you?’ rather than, ‘What is wrong with you?’. Threats to the authenticity of an individual's identity, as well as to their cues of safety, are at the core of trauma experiences. Hence, a focus on psychological safety may promote the shift to trauma-informed approaches that may be able to guarantee that both at the physical and emotional levels, the patients and the personnel may feel safe [24]. The sense of feeling that one may take interpersonal risks in a safe environment is believed to represent a hallmark feature of psychological safety. Safe environments may be represented by situations where an individual thinks or feels that they will not be punished by other individuals for asking for help, where they think that they will not be excluded by other individuals (relatedness-denying), where they will not be questioned by other individuals about their ability to ameliorate their skills (competence-denying), or where they will not be addressed by other individuals through a frightening language (autonomy-denying). Overall, an individual has to be able to instinctively feel that their basic needs will not be impeded, in order to perceive the environment as safe for interpersonal risk-taking. When people's fundamental needs are impeded, they often suffer from maladaptive consequences including physical symptoms, depression, burnout, and negative affect [25]. However, few psychometric tools have taken into account the issue of psychological safety within clinical settings. Accordingly, only a limited number of studies have tried to address this topic. In order to develop a scale that was able to assess interpersonal dynamics in a therapy context, Veale et al. [26] validated the Therapeutic Environment Scales (TESS) that included a safety subscale defined as ‘feeling safe with others to express needs or to try out new behaviors’ [26]. Eighty one individuals recruited from three distinct contexts—a therapy community that used a psychodynamic approach, an inpatient unit with specialized personnel, and a unit for anxiety disorders with specialized personnel—participated in the validation of the TESS. Results revealed that the TESS showed valid and reliable psychometric properties. The services were found to differ significantly in terms of compassion, feeling safe, extinction and tolerance of harmful behaviors, inconsistency, and strong emotional expression, as well as positive reinforcement of members' act of courage [26]. According to latest studies investigating positive affect using neuroscience techniques, two distinct kinds of positive affect may exist. The first kind may be dopaminergic in nature and is associated with a drive/seeking system, while the other may be opiate/oxytocin mediated and is associated with a soothing-contentment system [27]. Through the administration of the Activation and Safe/Content Affect Scale (ASCAS) in a sample of 203 students, Gilbert et al. [28] captured three factors associated with positive affect: an activated positive affect factor (that may be mediated by dopamine), a relaxed positive affect factor, and a safeness and contentment positive affect factor (that may be mediated by opiate/oxytocin). Interestingly, in regard to depression, stress, anxiety, self-criticism, and insecure attachment, the third safeness and contentment positive affect factor showed the strongest negative correlations. Therefore, a better understanding of the distinct forms and roles of positive affect may help to clarify the link between positive feelings and wellbeing [28]. Early life experiences have been shown to exert a profound effect on the social, psychological, and physiological features of functioning and development. Recalling and remembering negative or positive early life relational experiences and how they relate to psychopathology tools is one field of research, and recall of parenting attitudes has been the subject of several self-report investigations. The Early Memories of Warmth and Safety Scale (EMWSS), developed by Richter et al. [29] in a group of 180 participants, was designed to support people retrieve their own internal positive feelings, emotions, and early life events. This is in contrast to the majority of self-report studies, which concentrate on the recall of parental behaviors. In a similar fashion to what was found with TESS, with respect to retrieval of parental behavior, retrieval of childhood positive emotional feelings was a more sensitive predictor of psychopathology [29]. Overall, despite the research efforts provided through the development of the TESS, ASCAS and the EMWSS, no validated psychometric tools exist regarding psychological safety as a unique core construct in the domain of a soothing-contentment system, promoting physiological, emotional and cognitive processes signaling safety, compassion for others and social engagement.

Since Gilbert et al. [28] captured three factors associated with positive affect: an activated positive affect factor (that may be mediated by dopamine), a relaxed positive affect factor, and a safeness and contentment positive affect factor (that may be mediated by opiate/oxytocin). However, no psychometric tool assessing psychological safety as a unique core construct in the domain of a soothing-contentment system, promoting physiological, emotional and cognitive processes signaling safety, compassion for others and social engagement has been developed. Hence, the Neuroception of Psychological Safety Scale (NPSS) has been developed by Morton et al. [30]. Bodily sensations, compassion, and social engagement are the three subscales that are included in the 29-item NPSS. The NPSS is a scale aimed at assessing psychological safety taking into account the psychological, social and physiological elements. The capacity to feel compassion as well as experiencing connection, empathy, care, and willingness to support are associated to the compassion subscale. The social engagement subscale is defined by the availability of individuals who can be trusted, that make oneself feel understood, welcomed, and cared for, as well as having the freedom to express oneself without fear of judgment. According to the items, social interactions are described as safe and the environment as not hazardous. The body's inner sensations during a restful state are captured by the bodily sensations subscale, which includes items related to a steady stomach, a regular heartbeat, and a regular breathing. The NPSS is neurophysiologically rooted in the polyvagal theory [[31], [32], [33]] which conceptualizes the reflexive detection of danger without awareness carried out by the brain [34] as neuroception [35]. According to the PVT, if a neuroception of safety [36] is detected, physiological, emotional and cognitive processes signaling safety are promoted and compassion for others [37] and social engagement [38] are fostered, supported by the ventral vagal parasympathetic pathway. Conversely, when a neuroception of danger or life threat is detected, fight/flight responses (supported by the sympathetic nervous system) or immobilization, death feigning or dissociation responses (supported by the dorsal vagal parasympathetic pathway) are implemented, respectively [39]. The original NPSS was validated in a non-clinical sample of 336 participants. Exploratory factor analysis (EFA) and inspection of the scree plot suggested a 3-factor solution (root mean square error of approximation (RMSEA) = 0.083 [90% confidence interval (CI): 0.080, 0.086], comparative fit index (CFI) = 0.80, Tucker-Lewis Index (TLI) = 0.78), that was confirmed through confirmatory factor analysis (CFA) (RMSEA = 0.058, standardized root mean squared residual (SRMR) = 0.062, CFI = 0.86 and TLI = 0.84). The total score of the NPSS shows a Cronbach's α of 0.95. The scale and its subscales show good internal reliability, as revealed by the subscales' Cronbach's α values of 0.92 for bodily sensations, 0.94 for compassion, and 0.93 for social engagement. In addition to promoting a wider understanding of relationship problems and mental health challenges, the NPSS may influence novel assessment methods for trauma therapies (e.g., compassion focused therapy). For instance, recording client histories about what it is needed for them to feel safe at the psychophysiological level, in addition to the use of the NPSS to better evaluate changes in the window of tolerance for autonomic arousal in patients who underwent traumatic events in their past [30].

The NPSS was originally developed in English. In view of the absence of a validated Italian tool with appropriate psychometric properties designed to assess psychological safety, our research is focused on the validation of the Italian version of the NPSS and on the examination of its psychometric properties [30]. Our results may provide clinical and research environments with a valuable tool where the assessment of psychological safety is needed [[40], [41], [42]]. At present, no other known translations of the NPSS are available in Italian or in any other language. Our study aims to validate the NPSS analyzing the following: (a) content and face validity of the NPSS; (b) thorough psychometric properties and overall factorial structure; (c) the convergent validity of the NPSS, using the Self Compassion Scale-Short Form (SCS-SF [43]; Italian version in Poli et al. Submitted) and the Unconditional Self-Kindness Scale (USKS [44]; Italian version in Poli et al. Submitted), as well as its internal consistency; (d) the discriminant validity of the NPSS using the Body Perception Questionnaire-22 (BPQ-22 [45]; Italian version in Poli et al. [46]) and the Subjective Traumatic Outlook questionnaire (STO [47]; Italian version in Poli et al. Submitted); and (e) the reliability property of the NPSS by computing Spearman test-retest correlation, intraclass correlation coefficient, split-half Cronbach's αs, Spearman-Brown's coefficient and Guttman's Lambda 4 coefficient.

## 2 Materials and methods

### 2.1 Participants

Among the community members who responded to emails asking for volunteers to fill out psychological questionnaires, we recruited 338 (78.14% female) subjects (M = 42.56 years, SD = 10.38, years range 18–74). Regarding the participants' educational backgrounds, the highest percentage (78.92%) had a Ph.D. or specialization, followed by 16.56% with a higher level degree (master's or bachelor's degrees), and 4.52% with a medium level education (high school degree). The bulk of participants (82.07%) were actively working, 4.52% were undergraduate students and 13.41% of unoccupied, housewives or in retirement. Regarding marital status, 60.52% were married or cohabiting 29.48% of participants were single, 9.32% were divorced, and 0.68% were widowers or widows. In order to carry out subsequent statistical analyses, random split of the total sample generated a first random subsample consisting of 173 participants (75.15% female) that were used for EFA and exploratory structural equation modeling (ESEM), and a second random subsample consisting of 165 participants (81.21% female) that were used for CFA. A subsample of 127 participants (70.08% female) filled out the NPSS again after 3 weeks and were used for reliability analysis.

### 2.2 Measures

*Neuroception of Psychological Safety Scale (NPSS,* [30]*)*. The NPSS is a self-report tool developed by Morton et al. [30] to evaluate neuroception of psychological safety. Items ask respondents to indicate how much they agree with the assertions done in each of the 29 items on a 5-point scale (from 1 = “*strongly disagree*” to 5 = “*strongly agree*”). The NPSS showed a three-factor structure with a social engagement (SE) subscale (e.g., “There was someone who made me feel safe”), a compassion (COM) subscale (e.g., “I felt compassion for others”) and a bodily sensations (BS) subscale (e.g., “My breathing was steady").

The Italian version of the NPSS was completed by a mix of forward and reverse translation [48]. The writers and one multilingual (English and Italian) psychologist independently translated the scale's English version into Italian. Without any prior knowledge of the source language, an Italian-English researcher subsequently translated our Italian-translated version back into English, when the translators reached agreement. We examined the discrepancies in back translation with the scale's creators. Prior to being utilized in this study, our version of the NPSS translated in Italian was administered to ten individuals (who are not involved in this research) to evaluate the items' readability. It was established that every question was easy to understand and score. Regarding Cronbach's alpha (α, [49]) research has shown that a very high index (i.e., >0.95) may be inappropriate when constructing a test [50], although several reports exist on the adequate values, which range from 0.70 to 0.95 [[51], [52], [53]]. In our investigation, NPSS showed an α = 0.922, while the SE, COM, and BS subscales showed αs of 0.913, 0.847, and 0.906, respectively.

*Body Perception Questionnaire-22 (BPQ-22,* [45]*)*. The Body Perception Questionnaire (BPQ) was initially created by Porges [54] but later improved by Cabrera et al. [45] and Poli et al. [46] as a self-report test of body awareness and autonomic reactivity. In our study we used the 22-item Italian version [46]. Participants are asked to rate the frequency with which they feel aware of physical sensations (body awareness subscale (BOA), for example, “Watering or tearing of my eyes”), as well as the frequency of the supradiaphragmatic reactivity they experienced (supradiaphragmatic subscale (SUP), for example, " When I am eating, I have difficulty talking ") and subdiaphragmatic reactivity (subdiaphragmatic and body awareness subscale (BOA/SUB), for example, " After eating I have digestive problems ") on a 3-point scale (from 1 = *never* to 3 = *often*). The subscales for BOA, SUP, and BOA/SUB showed α values of 0.866, 0.815, and 0.812, respectively.

*Self-Compassion Scale-Short Form (SCS-SF,* [43]*)*. The ability to retain one's sensations of suffering with a sense of warmth, connection, and care is assessed by the SCS-SF, a measure of self-compassion [43]. Self-judgment, common humanity, mindfulness, self-kindness, isolation, and overidentification subscales are the six subscales that are included by the 12-item SCS-SF. On a 5-point rating scale (from 1 = *almost never* to 5 = *almost always*), participants are asked to evaluate how they typically treat themselves throughout difficulties (for example, " When I fail at something important to me I become consumed by feelings of inadequacy " or " When something upsets me I try to keep my emotions in balance "). Our study administered the Italian translation by Poli et al. (Submitted). The SCS-SF revealed an α = 0.869 in our investigation.

*Unconditional Self-Kindness Scale (USKS,* [44]*)*. Smith et al. [44] developed the USKS, a self-report tool designed to measure unconditional self-kindness. Using a 7-point rating scale (from 0 = *never* to 6 = *a great deal*), each of the six items asks participants to rate how much they agree with each of the questions (e.g., “How much are you loving and kind to yourself when you become aware of your personal flaws and imperfections?”, “How much are you patient and tolerant with yourself when you are criticized or rejected by another person?”). The translation by Poli et al. (Submitted) into Italian was administered in our investigation. A value of α = 0.923 was shown for the USKS.

*Subjective Traumatic Outlook questionnaire (STO,* [47]*)*. The STO consists of five items that participants are asked to score on a five-point rating scale (from 1 = *not at all* to 5 = *very much*) regarding how much each statement is true for themselves when recalling the worst or traumatic event in their life (e.g., " Looking on your condition, do you feel that you suffer from psychological trauma?”, “Do you feel that since the traumatic event no one can understand what you are going through?”). In our investigation we administered the Italian translation by Poli et al. (Submitted). The STO revealed an α = 0.87 in our study.

### 2.3 Procedure

A secure online survey tool (SurveyMonkey) was used to make the questionnaires accessible on the web. It took between 15 and 25 min to fill out the battery of questionnaires. A balanced approach was used to distribute the questionnaires in order to take order and sequence effects into consideration. The first sequence of administration was: NPSS, BPQ-22, SCS-SF, USKS, STO. The second sequence of administration was: STO, USKS, SCS-SF, BPQ-22, NPSS. Half of the participants were administered questionnaires with sequence one, while the other half with sequence two. Participants were randomly assigned to sequence one and sequence two. Following a thorough description of the study's methodology, all individuals accepted to participate, and were handled in accordance with the Ethical Principles of Psychologists and Code of Conduct [55]. No compensation for participation in this study was provided.

### 2.4 Statistical analyses

JASP 0.17.1 [56], SPSS® 27 (IBM Corp., Armonk, NY, USA), and Mplus 8.8 [57,58] were used to carry out the statistical analyses. As a first step, in order to determine if the distributions were normal, the Shapiro-Wilk test was used [59]. We calculated the data matrix determinant, the Kaiser-Meyer-Olkin (KMO) test for sample adequacy, and Bartlett's test of sphericity as factorability metrics. Within the dataset, the presence of redundant information (item pairs with very high levels of association) and items with low squared multiple correlations (SMC) were next evaluated. These factors, also referred to as “bloated specifics” ([60], p. 288), are usually generated by highly associated items that are frequently characterized by extremely similar topic and/or wording. When carrying out a factor analysis, these items could yield factors of minor importance. If an item's intercorrelation was more than 50% of the shared variance, or stronger than |0.707|, it was deemed redundant. Subsequently, the SMCs of the residual items were examined. When assessing initial communality—that is, the percentage of variation that each item's common factors account for—EFA software often use SMC—that is, the proportion of variance that each item shares with the others. Items with SMC lower than 0.10 should be eliminated from the group of items, as they are not expected to significantly affect the model [61].

Therefore, a random sample split was used. Prior to performing EFA on the first random subsample, we determined the optimal number of factors to be extracted using dimensionality metrics such as the scree-test [62], the parallel analysis (PA, [63]), and the minimal average partial (MAP) correlation statistic [64]. The decreasing eigenvalues curve begins to flatten out as the amount of factors rises. The point where the inflection point of the eigenvalues' curve is located, as determined by the scree-test, represents the optimal number of factors [62]. We used 1000 random correlation matrices generated by permuting the raw data to conduct PA in accordance with Buja and Eyuboglu's [65] guidelines. According to Longman et al. [66], the random-derived eigenvalues determined from the 95th percentile were used as threshold values. According to Velicer [64], after partialling out the factors, the average partial correlation of the variables—that is, the MAP statistic—is at its lowest value, indicating that the number of factors is ideal.

We used ESEM [67] to assess how well the models fit the data after the optimal number of factors had been determined. We used theta parameterization, GEOMIN rotation, and weighted least squares with means and variance adjustment (WLSMV) estimation. The definitive model was identified by using the same standards as previously stated for the CFA, taking into account the goodness-of-fit (GOF) indices and the most accurate approximation of a basic structure. We evaluated as significant the loadings whose 95% confidence interval was fully above the |0.32| threshold, taking into account the opportunity provided by ESEM to estimate the standard errors of loadings. After determining the measurement model using ESEM, then we used CFA to assess its fit. The one-factor, bi-factor, four-factor, and five-factor models were assessed in addition to the three-factor model.

Next, we used CFA and the WLSMV estimator (theta parameterization) to investigate the validity of the suggested three-factor structure on the second random subsample. The PClose, TLI, RMSEA with a 90% confidence interval, and CFI were used to assess the GOF. We used the following recommendations [68] to assess the model fit: an excellent fit was indicated by TLI and CFI values ≥ 0.95, an acceptable fit by TLI and CFI values ≥ 0.90; an excellent fit was suggested by RMSEA values < 0.06, an acceptable fit by RMSEA values ≤ 0.08. The PClose is a measure of the p-value considering the null hypothesis that the RMSEA estimate is actually below .05. Hence, with a PClose ≥0.05 the null hypothesis that the RMSEA is actually below 0.05 is not rejectable, and the fit of the model is confirmed.

Spearman correlations were computed between the additional questionnaires administered to the participants and the NPSS measures in order to investigate construct validity. The correlations ranging from 0.70 to 0.89 were deemed to be very strong, those ranging from 0.50 to 0.69 were deemed to be strong, those ranging from 0.30 to 0.49 were deemed to be moderate, and those ranging from 0.10 to 0.29 were deemed to be weak, in line with Cohen's [69] recommendations.

The scales were retested on a second sample of participants as an initial phase in determining reliability. The Spearman correlation coefficient was computed between the measures obtained at time 1 and after three weeks (time 2) as a retest coefficient. At time 2 we recruited 112 participants that also responded at time 1. The intraclass correlation coefficient (ICC) was used to analyze the steadiness of NPSS measures. Retest scores over 0.70 — that is, 50% or more of the shared variance — were deemed to show acceptable coefficients of stability. Specifically, with respect to ICC, we adhered to the cautious recommendations provided by Portney and Watkins [70]: coefficients ranging from 0.5 to 0.75 are deemed to be “poor to moderate,” whereas coefficients ranging from 0.75 to 0.9 are deemed to be “good.” We estimated the 95% confidence intervals whenever available. After using the split-half approach to examine the NPSS's reliability, the Spearman-Brown coefficient, and the Guttman's Lambda 4 coefficient, and the two-part Cronbach's α were computed [71,72] as further reliability indicators.

Inter-rater reliability was evaluated using Cohen's K statistics in order to evaluate content validity [73]. Rater reliability is substantial since it describes the degree to which the information obtained in our research are accurate depiction of the measures being examined. Two separate researchers (M.M. and A.P.) rated the NPSS items. To calculate the inter-rater consensus, Cohen's K statistics were computed. Following the criteria outlined by Fleiss, Levin, and Paik [74] and Cicchetti [75], the *K* statistics value was analyzed to determine whether it is excellent (higher than 0.74), good (ranging from 0.60 to 0.74), or fair (ranging from 0.40 to 0.59). The coefficient in our study was found to be excellent (*k* = 0.84). Ten students were used as convenience sample in order to assess the face validity of [76]. Participants were asked to provide a clearer phrasing for any questions they thought were confusing. The research team then debated the comments until an agreement was achieved and the tool's final version was developed.

## 3 Results

Factorability values were found to be acceptable for conducting exploratory analyses (determinant = 0.008; Bartlett's test of sphericity: degrees of freedom (df) = 406, χ2 = 5642.214, p < .0001; KMO test = 0.913). As we had to investigate the most appropriate model for the Italian NPSS without no prior information guiding our investigation, in order to identify a factor structure that may satisfy the conditions of an estimation of a simple structure, we chose to carry out an EFA on the first random subsample [61,77]. As a next step, we conducted a CFA on the second random subsample. Nevertheless, before doing these analyses, we first examined the whole dataset to find redundant items and those with low SMC. If the intercorrelation of an item was more than |0.707|, or over 50% of the variation shared between them, it was considered redundant. Not a single item exceeded this criterion. SMCs less than 0.10 indicate that an item is unlikely to significantly contribute to the measurement model, and hence, it may be eliminated from the item pool [61]. There was not a single item that demonstrated an SMC below this limit.

**Fig. 1:**
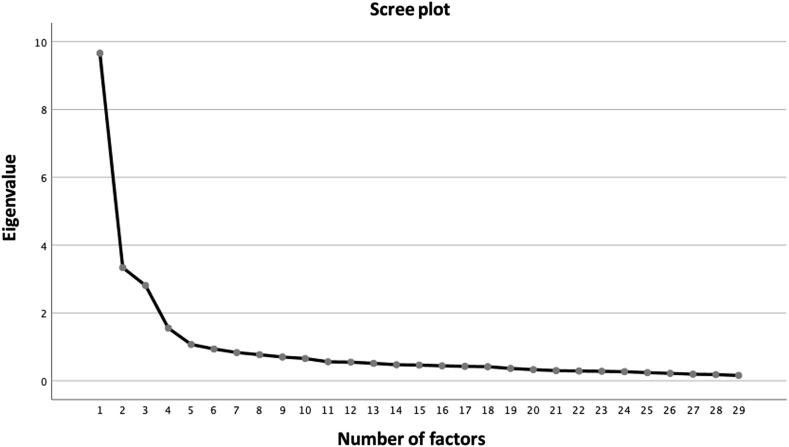
Results of the dimensionality analyses on the first random subsample (n = 173).

On the first random subsample, we performed dimensionality investigations using the scree-test [62], the PA [63], and the MAP correlation statistic [64]. The PA indicated that five of the observed eigenvalues exceeded the 95th percentile of the corresponding random eigenvalues. The scree-plot line seemed to flatten at the fifth factor, indicating that up to five factors may have been extracted (Fig. 1). Yet, the first element was where the MAP statistic fell the most short (0.0112, 0.0111, 0.0110, 0.0127, 0.0123, 0.0114). It was therefore clear that a maximum of three, four or five factors may be appropriate.

The fit of these models was then evaluated using ESEM [67] through WLSMV estimation, GEOMIN rotation, and theta parameterization. Table S1 reports the outcomes of the ESEM models. The one-factor and bi-factor ESEM model had a poor fit (one-factor: df = 223, χ^2^ = 595.353, CFI = 0.731, TLI = 0.733, RMSEA = 0.082 [0.081; 0.087]; bi-factor: df = 217, χ^2^ = 581.281, CFI = 0.865, TLI = 0.867, RMSEA = 0.055 [0.059; 0.057]). The four-factor and the five-factor ESEM model showed a somewhat greater fit (four-factor: df = 200, χ^2^ = 331.428, CFI = 0.958, TLI = 0.947, RMSEA = 0.032 [0.029; 0.035]; five-factor: df = 181, χ^2^ = 250.335, CFI = 0.978, TLI = 0.971, RMSEA = 0.025 [0.021; 0.029]), however, no item had a single loading in the first factor completely above the 0.32 confidence interval. The three-factor approach suited the data adequately (df = 211, χ^2^ = 387.521, CFI = 0.945, TLI = 0.934, RMSEA = 0.035 [0.032; 0.039]) and it was determined to be the most accurate measurement model as each factor had a minimum of six items with a single loading and a confidence interval that was completely above 0.32. A three-factor model was found to be loaded by the 29 items (Table 1).

**Table 1:** Loading matrix and factor correlations of the three-factor Exploratory Structural Equation Modeling solution on the first random subsample (n = 173).

Item	F1	F2	F3
NPSS01	0.68 [0.22; 0.46]	0.28 [0.24; 0.03]	0.19 [0.18; 0.47]
NPSS02	0.62 [0.23; 0.46]	0.38 [0.21; 0.08]	0.20 [0.12; 0.42]
NPSS03	**0.61 [0.32; 0.51]**	0.28 [0.02; 0.28]	0.20 [0.07; 0.36]
NPSS04	**0.73 [0.37; 0.58]**	0.27 [0.06; 0.24]	0.10 [0.04; 0.27]
NPSS05	**0.68 [0.48; 0.68]**	0.31 [0.13; 0.16]	0.07 [0.02; 0.29]
NPSS06	0.66 [0.27; 0.51]	0.33 [0.17; 0.42]	0.11 [0.21; 0.04]
NPSS07	0.61 [0.12; 0.26]	−0.01 [0.06; 0.09]	0.14 [0.23; 0.03]
NPSS08	0.64 [0.26; 0.49]	0.07 [0.11; 0.37]	0.20 [0.08; 0.11]
NPSS09	0.76 [0.23; 0.48]	0.11 [0.18; 0.43]	0.14 [0.09; 0.19]
NPSS10	0.72 [0.12; 0.18]	0.18 [0.32; 0.57]	0.07 [0.11; 0.14]
NPSS11	**0.78 [0.41; 0.64]**	0.11 [0.07; 0.34]	0.12 [0.25; 0.02]
NPSS12	**0.80 [0.42; 0.66]**	0.04 [0.03; 0.09]	0.15 [0.24; 0.03]
NPSS13	**0.61 [0.36; 0.50]**	0.16 [0.13; 0.19]	0.12 [0.16; 0.08]
NPSS14	**0.67 [0.41; 0.60]**	0.17 [0.11; 0.16]	0.08 [0.12; 0.14]
NPSS15	0.28 [0.18; 0.29]	0.17 [0.15; 0.10]	**0.68 [0.33; 0.44]**
NPSS16	0.20 [0.17; 0.30]	0.26 [0.15; 0.25]	0.66 [0.31; 0.43]
NPSS17	0.10 [0.08; 0.20]	−0.35 [-0.12; 0.23]	**0.66 [0.32; 0.44]**
NPSS18	0.06 [0.07; 0.15]	0.09 [0.08; 0.13]	**0.75 [0.41; 0.68]**
NPSS19	0.18 [0.14; 0.23]	0.03 [0.05; 0.18]	**0.56 [0.33; 0.45]**
NPSS20	0.14 [0.13; 0.25]	0.10 [0.18; 0.25]	**0.79 [0.44; 0.66]**
NPSS21	0.13 [0.10; 0.17]	0.09 [0.17; 0.26]	**0.80 [0.43; 0.70]**
NPSS22	0.18 [0.18; 0.23]	**0.64 [0.38; 0.45]**	0.14 [0.09; 0.20]
NPSS23	0.06 [0.07; 0.10]	**0.75 [0.43; 0.67]**	0.10 [0.15; 0.25]
NPSS24	0.21 [0.19; 0.33]	**0.71 [0.49; 0.69]**	0.06 [0.01; 0.12]
NPSS25	0.19 [0.24; 0.33]	**0.72 [0.33; 0.58]**	0.13 [0.19; 0.21]
NPSS26	0.16 [0.12; 0.27]	**0.71 [0.34; 0.59]**	0.04 [0.07; 0.15]
NPSS27	0.14 [0.16; 0.28]	**0.81 [0.46; 0.77]**	0.08 [0.14; 0.23]
NPSS28	0.17 [0.09; 0.17]	**0.68 [0.37; 0.44]**	−0.01 [0.05; 0.07]
NPSS29	0.23 [0.24; 0.31]	0.68 [0.31; 0.43]	0.10 [0.12; 0.24]

Note: Bracketed values and the 95% confidence interval of the loading estimate. Bolded values indicate that this interval is entirely over |0.32|.

**Fig. 2:**
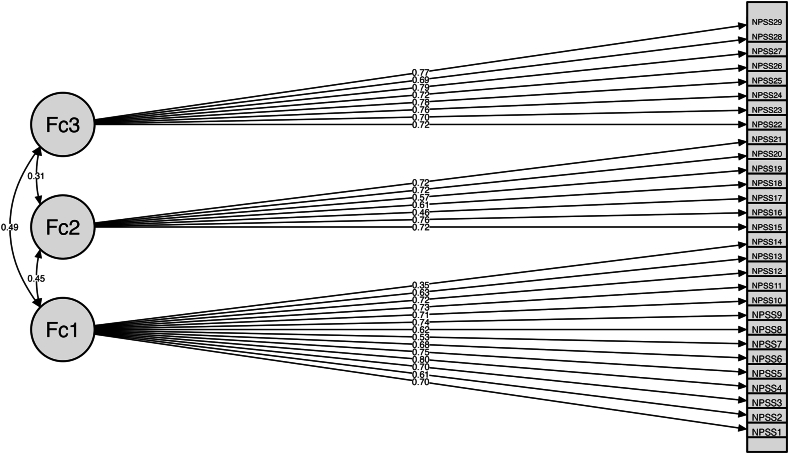
Confirmatory Factor Analysis model for the three-factor solution (n = 165). Values above arrows indicate factor loadings. NPSS = Neuroception of Psychological Safety Scale item; Fc = NPSS factor.

The validity of the proposed three-factor, bi-factor or four-factor structures was then examined on the second random subsample using CFA and the WLSMV estimator (Table 2). The findings showed a poor fit for the bi-factor (df = 184, χ^2^ = 600.137, CFI = 0.921, TLI = 0.914, RMSEA = 0.082 [0.077; 0.087], PCLOSE <0.001) and the four-factor model (df = 181, χ^2^ = 422.493, CFI = 0.954, TLI = 0.950, RMSEA = 0.063 [0.057; 0.068], PCLOSE <0.001). The three-factor model confirmed an appropriate fit (df = 182, χ^2^ = 266.957, CFI = 0.984, TLI = 0.983, RMSEA = 0.037 [00.030; 0.044], PCLOSE = 0.874) (Fig. 2) and factor loadings (Table S2).

**Table 2:** Goodness-of-fit indices for the Confirmatory Factor Analyses on the second random subsample (n = 165).

Model	χ^2^	df	CFI	TLI	RMSEA [90% CI]	PCLOSE
Bifactor	600.137	184	0.921	0.914	0.082 [0.077; 0.087]	<0.001
Three-factor	266.957	182	0.984	0.983	0.037 [0.030; 0.044]	0.874
Four-factor	422.493	181	0.954	0.950	0.063 [0.057; 0.068]	<0.001

Note: all chi-square tests were significant at *p* < 0.001; df = degrees of freedom; CFI = Comparative Fit Index; TLI = Tucker Lewis Index; RMSEA = Root Mean Square Error of Approximation; CI = confidence interval.

The associations between the NPSS scores and the other scales in this research are shown in Table 3. The NPSS translated into Italian (Table S3) revealed quite comparable, positive moderate associations with the SCS-SF and the USKS, indicating that higher scores on self-compassion and unconditional self-kindness are linked to an increased propensity to perceive a psychological neuroception of safety, confirming the scale's convergent validity. The NPSS scores, on the other hand, were found to have a negative weak correlation with the BOA, SUP and BOA/SUB subscales of the BPQ-22 and with the STO questionnaire, indicating that higher scores on the neuroception of psychological safety are linked to a lower propensity to consciously perceive negative bodily feelings and to report traumatic experiences. Overall, these results bolster the NPSS's discriminant and convergent validity.

**Table 3:** Spearman correlations among NPSS and the other study measures (n = 338).

Scales	1	2	3	4	5	6	7	8	9	10
1. NPSS	0*.922*									
2. SE	0.866***	0*.913*								
3. COM	0.608***	0.374***	0*.847*							
4. BS	0.726***	0.417***	0.276***	0*.906*						
5. BOA	−0.103*	−0.055	−0.020	−0.159**	0*.866*					
6. SUP	−0.234***	−0.094	−0.120*	−0.373***	0.224***	0*.815*				
7. BOA/SUB	−0.146**	−0.055	−0.004	−0.266***	0.431***	0.359***	0*.812*			
8. SCS-SF	0.481***	0.383***	0.271***	0.422***	−0.250***	−0.214***	−213***	0*.869*		
9. USKS	0.376***	0.313***	0.219***	0.307***	−0.173**	−0.154**	−0.162**	0.723***	0*.923*	
10. STO	−0.283***	−0.249***	−0.062	−0.331***	0.191***	0.147**	0.225***	−0.358***	−0.183***	0*.870*
M	112.044	51.074	29.305	31.666	11.396	9.627	12.154	40.346	20.089	10.586
SD	15.316	9.235	4.322	6.048	3.755	2.477	3.445	8.824	6.863	5.182
Median	113.5	51	30	32	10	9	12	41	20	10
IQR	19	10.75	6	10	5	2	5	12	9	9

Note: ***: correlations are significant at *p* < 0.001. Italicized values on the main diagonal are Cronbach's alphas. NPSS: Neuroception of Psychological Safety Scale-Generic version; SE: Social Engagement subscale of the NPSS; COM: Compassion subscale of the NPSS; BS: Bodily Sensations subscale of the NPSS; BOA: Body Awareness subscale of the Body Perception Questionnaire-22 (BPQ-22); SUP: Supradiaphragmatic reactivity subscale of the Body Perception Questionnaire-22 (BPQ-22); BOA/SUB: Body Awareness/Subdiaphragmatic reactivity subscale of the Body Perception Questionnaire-22 (BPQ-22); SCS-SF: Self-Compassion Scale – Short Form; USKS: Unconditional Self-Kindness Scale; STO: Subjective Traumatic Outlook questionnaire; M: mean; DS: standard deviation; IQR: Interquartile Range.

Using a different participant sample, we next examined the scale's test-retest reliability. According to Table 4, which summarizes the findings, the test-retest correlation was 0.78, indicating that the scores were reasonably consistent across a three-week period. The intraclass correlation coefficient (ICC) test was also performed, and the results confirmed that the scores were consistent over a three-week period (ICC = 0.922). Additionally, we used the split-half method to evaluate reliability and discovered the two split-half Cronbach's αs to be appropriate (split-half 1's = 0.913; split-half 2's = 0.868), along with the Spearman-Brown coefficient (C_SB_ = 0.71) and the Guttman's Lambda 4 coefficient (G_L4_ = 0.72).

**Table 4:** Reliability measures for the NPSS scale.

Reliability measure	Test		Retest			
Test-retest	M	SD	M	SD	ρ_tt_	ICC
	55.43	9.56	56.61	7.90	0.78***	0.922***
Split-half	Split-half-1 – Cronbach's α		Split-half-2 – Cronbach's α		C_SB_	G_L4_
	0.913		0.868		0.71***	0.72

Note: NPSS: Neuroception of Psychological Safety Scale-Generic version; M: mean; SD: standard deviation; ρ_tt_: Spearman test-retest correlation; ICC: Intraclass correlation coefficient; α = standardized Cronbach's α; C_SB_ = Spearman-Brown's coefficient; G_L4_ = Guttman's Lambda 4 coefficient; ***: *p* < 0.001.

Eventually, Cohen's *k* coefficient in our study was found to be excellent (k = 0.84).

## 4 Discussion

The aim of the present investigation was to validate the Italian version of the NPSS [30], assessing its factor structure, content, convergent, discriminant, and face validity, and reliability over time. Our findings showed that content, convergent, discriminant and face validity, as well as reliability over time were supported. Regarding content validity, following the criteria outlined by Fleiss, Levin, and Paik [73] and Cicchetti [74], we found that the value for *K* statistics was shown to be excellent (*k* = 0.84). Consistent with the original validation by Morton et al. [30], our data suggested a three-factor structure. The inflection point on the scree-plot advised that three factors may be extracted; however, in ESEM we also tested one-factor, bi-factor, four-factor and five-factor solution. Model fit of a bifactor solution in ESEM may be expected to show a better fit than a three-factor structure. Nonetheless, many investigations indicated the parameters' fit values for various models. In their study, Yi et al. [78] investigated the fit of an ESEM model including two or three distinct factors. Their results indicate that a three-factor representation yields an improved model fit. Furthermore, the CFI and TLI enhanced while the χ2/df ratio and RMSEA reduced when the number of unique factors varied from two to three, two to six, three to four, or three to six, according to average estimations among reports. Interestingly, this pattern is reflected in our ESEM models from two to three factors. In particular, CFI and TLI changed from 0.865 to 0.867 to 0.945 and 0.934, respectively; while χ2/df ratio and RMSEA changed from 2.68 to 0.055 to 1.84 and 0.035, respectively [79]. Regarding newly generated items, saturation per item is recommended to be > 0.5, whereas for existing items, saturation for each item is recommended to be ≥ 0.6 [80]. Our findings meet these requirements since all factor loadings are greater than 0.61. According to the criteria established by Marsh et al. [68], the CFA results revealed an adequate fit for the three-factor model.

We discovered that the NPSS and the USKS scores as well as the NPSS and the SCS-SF scores were found to show comparable moderate positive Spearman correlations in order to assess convergent validity. Wouters-Soomers et al. [25] examined what is required in order to develop psychological safety at the individual level. Their findings demonstrated that for people to develop the meaningful relationships that foster psychological safety, they either need to have their fundamental needs met or show self-compassion. In addition, in order to measure, and enhance, psychological safety in mental health services, team-level surveys are administered that comprises proposed indicators of psychological safety as perceived compassion, as well as perceived institutional and managerial help [81]. Finally, it has been shown that among the key-points for ensuring a psychologically safe learning environment in a clinical context kindness represents a fundamental component [82]. Taken together, it may be hypothesized that these findings indicate that in order to develop, and maintain, psychological safety both self-compassion and self-kindness may be needed. Interestingly, it has been shown that loving-kindness may imply intentionally cultivating happiness and may result in a direct emotional experience that is dependent upon the activation of the reward system of the brain, the mesolimbic dopaminergic circuit [83]. Analogously, it has been hypothesized that compassion stimulates the brain's positive emotion systems [84]. Overall, a focus on the activation of the positive affect system may be needed in order to foster and cultivate psychological safety.

Regarding discriminant validity, consistent with the previous findings, a weak negative correlation was revealed between NPSS and the STO questionnaire, highlighting the fact that when traumatic feelings are acutely active in the body feelings of psychological safety are difficult to be elicited. Regarding Spearman correlations between NPSS and BPQ-22 subscales, we found negative weak correlations among NPSS and the three subscales of the BPQ-22, BOA, SUP and BOA/SUB. At first glance, it may seem a surprising result, however, inspecting the items of the BPQ-22, the wording of the items is biased towards a representation of unpleasant bodily feelings. Hence, in order to be able to feel psychological safety it may be needed that feelings of unpleasant bodily sensations are at low levels. In accordance with this, it has been shown that those who have a healthy body image and higher levels of body trusting also show higher levels of psychological safety and would be more resilient and able to handle stress and adversity, which will lead to sustained work performance [85]. In addition, in the context of interpersonal interactions, body trusting may improve work performance. In a community, pleasant behavior is encouraged and enhanced communication is made possible by psychological safety that results from body trusting [85]. Interestingly, mindfulness and self-reassurance have been shown to be able to reduce the neural activity of the negative affect system [84,86], and, therefore, they may be useful at reducing bodily traumatic feelings. Taken together, in order to develop, and maintain a sense of psychological safety, self-compassion and self-kindness may promote the activity positive affect system and, synergistically, self-reassurance and mindfulness [87], may foster a decrease in the negative affect system and protect the individual from bodily traumatic feelings.

We assessed the test-retest reliability of the NPSS using data from a different sample of individuals who provided their answers three weeks later. Over a 3-week period, the NPSS scores were constant; in fact, the Spearman test-retest correlation was higher than 0.70, and the ICC was revealed to be above 0.80 in accordance with the conservative standards proposed by Portney and Watkins [70]. These findings suggested that the NPSS had a good test-retest reliability. In addition, utilizing a split-half approach, it was discovered that the Guttman's Lambda 4 coefficient, the Spearman-Brown's coefficient, and the two split-half Cronbach's alpha were higher than 0.71, indicating the good test-retest reliability of the NPSS.

## 5 Conclusions

The following limitations should be taken into consideration when interpreting the results of this study: a) from the Italian population a relatively huge non-clinical sample was recruited and the psychometric properties of the scale were initially investigated; additional research is needed to validate the scale's three-factor structure and sufficient validity and reliability in clinical samples; b) the participants' demographics were not typical of the general population, which might restrict how broadly the findings can be applied; c) three of the research's measures do not currently possess a published validation in the Italian language, as the papers are either being prepared for publication or are still in the publishing process; and d) criterion and nomological validity were not assessed, which should be evaluated in subsequent studies.

Our research offered a first indication that the NPSS is a valid and reliable three-dimensional scale to measure the neuroception of psychological safety. The NPSS is a psychometric tool assessing psychological safety as a unique core construct in the domain of a soothing-contentment system, promoting physiological, emotional and cognitive processes signaling safety, compassion for others and social engagement. Rooted in the PVT, psychological safety is promoted by a neuroception of safety that, in turn, is neurophysiologically supported by the ventral vagal parasympathetic pathway. Before the tool may be confidently used in research and clinical situations where this construct is relevant, further studies are necessary to verify the tool in languages other than Italian and to confirm these findings.

## Compliance with ethical standards

All procedures performed in research involving human participants were according to the institutional (Bioethics Committee, protocol code 0036344/2020) and 1964 Helsinki declaration and its subsequent amendments.

## Data availability statement

The datasets generated during and/or analyzed during the current study are available from the corresponding author on reasonable request.

## Informed consent statement

Informed consent has been obtained from all the subjects involved in the study.

## Funding

This research received no external funding.

## CRediT authorship contribution statement

**Andrea Poli:** Writing – review & editing, Writing – original draft, Validation, Software, Resources, Methodology, Investigation, Formal analysis, Data curation, Conceptualization. **Mario Miccoli:** Writing – review & editing, Software, Methodology, Data curation, Conceptualization.

## Declaration of competing interest

The authors declare the following financial interests/personal relationships which may be considered as potential competing interests: Andrea Poli reports a relationship with Cell Press that includes: board membership.
