# Supplementary material for: Validation of the Italian version of the Neuroception of Psychological Safety Scale (NPSS)
Source: Heliyon. 2024 Mar 16;10(6):e27625. doi: 10.1016/j.heliyon.2024.e27625 (PMC10963227; doi:10.1016/j.heliyon.2024.e27625)
Supplement: Multimedia component 1 [file mmc1.pdf]

QuillBot

Scanned on: 10:28 March 16, 2023 UTC

0.0%	0	5050
Overall similarity score	Results found	Total words in text

	Word count
Identical	0
Minor Changes	0
Omitted	0

Powered by COPYLEAKS

Results

The results include any sources we have found in your submitted document that includes the following: identical text, minor changed text, paraphrased text.

No results found. The content is free of plagiarism.

IDENTICAL

Text that is exactly the same.

MINOR CHANGES

Text that is nearly identical, yet a different form of the word is used. (i.e 'slow' becomes 'slowly')

Unsure about your report?

The results have been found after comparing your submitted text to online sources, open databases and the Copyleaks internal database. If you have any questions or concerns, please feel free to contact us atsupport@copyleaks.com

Click here to learn more about different types of plagiarism

QuillBot

QuillBot

Scanned on: 10:28 March 16, 2023 UTC

Scanned Text

Your text is highlighted according to the plagiarism types that where found, as shown above.

IDENTICAL MINOR CHANGES

1. Introduction
Danger perception and, in particular, perceiving one’s own life threatened, is believed to represent a major predictor of posttraumatic stress disorder (PTSD) after experiencing a traumatic injury [1], as well as a maintenance factor [2], and persistent danger perception may lead to hypervigilance. Accordingly, compared to controls, children and adolescents with PTSD differentially directed processing resources away from depression-related stimuli and toward socially threatening stimuli [3]. Persistent danger perception is concurrently associated with a persistence of lack of safety, and research on the neuroscience of fear in both humans and non-humans has suggested that a lack of safety signal learning may be a biological hallmark of PTSD [4].

The Veterans Health Administration and Department of Defense, and the American Psychological Association released treatment guidelines for PTSD in 2017 that include a list of suggestions for healthcare professionals who are involved in the treatment of PTSD patients. Prolonged Exposure, Cognitive Processing Therapy, and trauma-focused Cognitive Behavioral Therapy (CBT) were all highly recommended. Each of these therapies has a strong body of research and they are trauma-focused, which means that these therapies deal with the traumatic event's memories or associated thoughts and emotions [5]. However, none of these therapies directly address the issue of safety feelings. Safety issues for PTSD were addressed by a specific form of CBT, named “Seeking Safety” [6]: the term referred to the fundamental principle of the therapy, which stated that in order to recover from PTSD safety had to be given priority. Safety was described as the creation of a support network and self-defense against risks related to the disease (e.g., domestic violence). Themes that were deemed to be most important for this patients were addressed in all sessions: for instance, seeking support, taking care of oneself, establishing boundaries [6]. Results of “Seeking Safety” treatment showed significant improvement for women and adolescent girls with PTSD and substance use disorder (SUD) [7,8] and in veterans with PTSD and SUD [9,10].

In healthcare work environments, the issues of psychological safety has been taken into account defining it as the possibility for workers to express themselves in a professional setting without worrying about shame or judgment from others [11]. This research line highlights that personal, team-based, or organizational elements may all affect psychological safety. While most of the prior research has been on the leaders' role in fostering psychological safety, a safe environment may be produced by any team member [11]. In addition, among emergency nurses, psychological safety has been found to represent a main factor potentially affecting their patient safety competency [12]. Accordingly, a composite measure, combining the advantages of observational and survey measurements, has been developed designed for use by healthcare teams [13]. Overall, a conceptual analysis regarding 88 articles investigating psychological safety in healthcare settings was able out to point out five factors critically contributing to psychological safety: solid interpersonal bonds, perception of the effects of interpersonal risk-taking, group-level phenomena, a safe work atmosphere where taking interpersonal risks is encouraged, and a non-punitive culture [14].

As highlighted by Sweeney et al. [15], organizational and mental health services need a paradigm shift from trauma-uninformed- to trauma-informed mental healthcare approaches in order to avoid re-traumatization of patients by trauma-uninformed staff, and to avoid the vicarious traumatization of staff by the patients’ traumatic expression and by practices like confinement and isolation. Threats to a person's safety, and often to the integrity of their identity, are at the core of trauma experiences. Hence, a

focus on psychological safety may promote the shift to trauma-informed approaches that may be able to guarantee that the staff members and the patients are both emotionally and physically safe [15]. However, few psychometric tools have taken into account the issue of psychological safety within clinical settings. In order to develop a scale that was able to assess interpersonal dynamics in a therapy context, Veale et al. [16] validated the Therapeutic Environment Scales (TESS) that included a safety subscale defined as 'feeling safe with others to express needs or to try out new behaviors' [16]. Through the administration of the Activation and Safe/Content Affect Scale, Gilbert et al. [17] captured three factors associated with positive affect: an activated positive affect factor, a relaxed positive affect factor, and a safeness and contentment positive affect factor. Interestingly, in regard to depression, stress, anxiety, self-criticism, and insecure attachment, the third safeness and contentment positive affect factor showed the strongest negative correlations [17]. In addition, the Early Memories of Warmth and Safety Scale was developed [18], which was related to retrieve one's own internal positive sensations, emotions, and events in childhood. In a similar fashion to what was found with TESS, with respect to retrieval of parental behavior, retrieval of childhood positive emotional feelings was a more sensitive predictor of psychopathology [18]. Overall, despite these research efforts, no validated Italian psychometric tools exist regarding psychological safety as the core construct.

Recently, in order to address these limitations, the Neuroception of Psychological Safety Scale (NPSS) has been developed by Morton et al. [19]. The NPSS is a 29-item tool with three subscales (compassion, social engagement, and bodily sensations) aimed at assessing psychological safety taking into account the psychological, social and physiological elements. The NPSS is neurophysiologically rooted in the polyvagal theory (PVT) [20,21] which conceptualizes the reflexive detection of danger without awareness carried out by the brain [22] as neuroception [23]. According to the PVT, if a neuroception of safety is detected, physiological, emotional and cognitive processes signaling safety are promoted and compassion for others [24] and social engagement [25] are fostered, supported by the ventral vagal parasympathetic pathway. Conversely, when a neuroception of danger or life threat is detected, fight/flight responses (supported by the sympathetic nervous system) or immobilization, death feigning or dissociation responses (supported by the dorsal vagal parasympathetic pathway) are implemented, respectively [26].

The aim of our article is to validate the Italian version of the NPSS and investigate its psychometric properties [19] to provide the availability of a useful tool that can be administered in research and clinical contexts [27–29], considering the lack of a validated Italian tool with sound psychometric properties aimed to assess psychological safety. Our study aims to validate the NPSS analyzing the following: (a) the NPSS face and content validity; (b) the factor structure and psychometric properties of the NPSS; (c) the NPSS internal consistency and its convergent validity with the Self Compassion Scale-Short Form (SCS-SF [30]; Italian version in Poli et al. Submitted), and with the Unconditional Self-Kindness Scale (USKS [31]; Italian version in Poli et al. Submitted); (d) the NPSS discriminant validity with the Body Perception Questionnaire-22 (BPQ-22 [32]; Italian version in Poli et al. [33]), the Subjective Traumatic Outlook questionnaire (STO [34]; Italian version in Poli et al. Submitted); and (e) the NPSS reliability by computing Spearman test-retest correlation, intraclass correlation coefficient, split-half Cronbach's αs, Spearman-Brown's coefficient and Guttman's Lambda 4 coefficient.

2. Materials and Methods

2.1. Participants

The sample included 338 (78.14% female) community members (M = 42.56 years, SD = 10.38 range 18-74) who replied to email requests for volunteers to complete psychological questionnaires. The participants' educational backgrounds were as follows: those with the greatest levels of education (Ph.D. or specialization) were 78.92%, 16.56% were found to have a higher level degree (master's or bachelor's degrees), and those who showed a medium level of education (high school degree) were 4.52%. The bulk of participants (82.07%) were actively working, 4.52% were undergraduate students and 13.41% of unoccupied, housewives or in retirement. Regarding marital status, 60.52% were married or cohabiting 29.48% of participants were single, 9.32% were divorced, and 0.68% were widowers or widows.

Measures

Neuroception of Psychological Safety Scale (NPSS, [19]). The NPSS is a self-report tool developed by Morton et al. [19] to evaluate neuroception of psychological safety. Items ask respondents to rate their level of agreement with the statements made in each of the 29 items on a 5-point scale (from 1 = "strongly disagree" to 5 = "strongly agree"). The NPSS showed a three-factor structure with a social engagement (SE) subscale (e.g., "There was someone who made me feel safe"), a compassion (COM) subscale (e.g., "I felt compassion for others") and a bodily sensations (BS) subscale (e.g., "My breathing was steady").

The Italian version of the NPSS was completed by a mix of forward and reverse translation [35]. The writers and one psychologist who is bilingual in Italian and English independently translated the scale's English version into Italian. An Italian-English researcher who was not familiar with the original language subsequently translated this Italian-translated version back into English, when the translators reached agreement. These back-translation differences were discussed with the scale's creators. The Italian version of the NPSS was administered to 10 persons (not included in the present research) in order to assess the readability of the items before being used in this research. It was established that every question was easy to understand and score. Regarding Cronbach's alpha (α, [36]) research has shown that a very high index (i.e., > 0.95) may be inappropriate when constructing a test [37], while there are many reports concerning the acceptable values ranging from 0.70 to 0.95 [38–40]. In our investigation, NPSS showed an α = 0.922,

while the SE subscale showed an α = 0.913, the COM subscale showed an α = 0.847 and the BS subscale showed an α = 0.906.
Body Perception Questionnaire-22 (BPQ-22, [32]). The Body Perception Questionnaire (BPQ) was initially developed by Porges [41] and later improved by Cabrera et al. [32] and Poli et al. [33] as a self-report test of body awareness and autonomic reactivity. In our study we used the 22-item Italian version [33]. Participants are asked to rate the frequency with which they feel aware of physical sensations (body awareness subscale (BOA), for example, "Watering or tearing of my eyes"), as well as the frequency with which they experience supradiaphragmatic reactivity (supradiaphragmatic subscale (SUP), for example, " When I am eating, I have difficulty talking ") and subdiaphragmatic reactivity (subdiaphragmatic and body awareness subscale (BOA/SUB), for example, " After eating I have digestive problems ") on a 3-point scale (from 1 = never to 3 = often). The BOA subscale showed an α = 0.866, the SUP subscale showed an α = 0.815 and the BOA/SUB subscale showed an α = 0.812.
Self-Compassion Scale-Short Form (SCS-SF, [30]). The ability to retain one's sensations of suffering with a sense of warmth, connection, and care is assessed by the SCS-SF, a measure of self-compassion [30]. The SCS-SF is a 12-item tool that comprises 6 subscales: self-judgment, common humanity, mindfulness, self-kindness, isolation, and overidentification. Participants are asked to rate on a 5-point scale (from 1 = almost never to 5 = almost always) how they usually treat themselves during challenges (for example, " When I fail at something important to me I become consumed by feelings of inadequacy" or " When something upsets me I try to keep my emotions in balance"). The Italian translation by Poli et al. (Submitted) was used in this investigation. In our study, the SCS-SF showed an α = 0.869.
Unconditional Self-Kindness Scale (USKS, [31]). Smith et al. [31] developed the USKS, a self-report tool designed to measure unconditional self-kindness. Participants are asked to score their degree of agreement with the issues posed in the six items (e.g., “How much are you loving and kind to yourself when you become aware of your personal flaws and imperfections?”, “How much are you patient and tolerant with yourself when you are criticized or rejected by another person?”) on a 7-point scale (from 0 = never to 6 = a great deal). In our study we used the Italian translation by Poli et al. (Submitted). The USKS was found to show an α = 0.923.
Subjective Traumatic Outlook questionnaire (STO,[34]). The STO is a list of 5 items that are aimed to ask the participants to rate on a 5-point scale (from 1 = not at all to 5 = very much) how much each statement is true for themselves when thinking back upon the most stressful or traumatic event in their life (e.g., " Looking on your condition, do you feel that you suffer from psychological trauma?", “Do you feel that since the traumatic event no one can understand what you are going through?”). The Italian version by Poli et al. (Submitted) was used in our study. The STO showed a α = 0.87.

2.2. Procedure

The questionnaires were made available online using a secure web-based survey program (SurveyMonkey). It took between 15 and 25 minutes to fill out the battery of questionnaires. A balanced approach was used to distribute the questionnaires in order to take order and sequence effects into consideration. All subjects, who volunteered to take part in the study after receiving a detailed explanation of the procedure, were treated in accordance with the Ethical Principles of Psychologists and Code of Conduct [42]. No compensation for participation in this study was provided.

2.3. Statistical analyses

SPSS® 27 (IBM Corp., Armonk, NY, USA), JASP 0.17.1 [43], and Mplus 8.8 [44,45] were used to carry out the statistical analyses. As a first step, the Shapiro-Wilk test was used [46] to verify whether the distributions were normal. As factorability metrics, we computed the data matrix determinant, Bartlett's test of sphericity, and Kaiser-Meyer-Olkin (KMO) test for sample adequacy. The occurrence of redundant information (item pairings that are very highly associated) and items with low squared multiple correlations (SMC) were then assessed using the dataset. Known as "bloated specifics" ([47], p. 288), these factors are often yielded by very strongly correlated items that are frequently characterized by extremely similar topic and/or wording. These items may generate factors of low importance in factor analysis. Items were considered redundant if their intercorrelation was stronger than |0.707|, or more than 50% of the shared variance. The SMCs of the residual items were then inspected. EFA software often uses SMC, or the proportion of variance that each item shares with the others, to evaluate initial communality, or the share of variation that each item's common factors account for. As they are unlikely to have a major impact on the measurement model, items with SMC smaller than 0.10 should be removed from the item pool [48]. Therefore, a random sample split was used. Using dimensionality metrics as the scree-test [49], the parallel analysis (PA, [50]), and the minimum average partial (MAP) correlation statistic [51], we first assessed the ideal number of components to be extracted before running EFA on the first random subsample. When the number of factors increases, the descending eigenvalues curve starts to flatten out. The optimal number of factors, according to the scree-test, corresponds to the point where the inflection point of the eigenvalues’ curve [49]. In a PA technique, the simulated eigenvalues from a random data matrix of the same size are compared to the observed eigenvalues. According to Buja and Eyuboglu's [52] recommendations, we performed PA using 1000 random correlation matrices obtained by permuting raw data. Longman et al. [53] suggest that the threshold values used were the 95th percentile random-derived eigenvalues. In accordance with Velicer [51], the number of factors is optimal when the average partial correlation of the variables (i.e., the MAP statistic) is at its lowest value, after partialling out the factors.
We used Exploratory structural equation modeling (ESEM, [54]) to assess how well the models fit the data

after the optimal number of factors had been determined. We employed weighted least squares with means and variance adjustment (WLSMV) estimation, theta parameterization, and GEOMIN rotation. Overall, ESEM allows for the investigation of complex factor structures (similar to EFA) and all factor loadings' estimates, while also allowing the analysis of goodness-of-fit (GOF) indices, parameter estimates, standard errors, and modeling flexibility, which are typical characteristics of confirmatory factor analysis (CFA). Using the same criteria as mentioned above for the CFA, the final model was selected based on the GOF indices and the best approximation of a simple structure. Considering the possibility afforded by ESEM to estimate the standard errors of loadings, we evaluated as significant those loadings whose 95% confidence interval was fully above the |0.32| criteria. After determining the measurement model by ESEM, we evaluated its fit through CFA. In addition to the three-factor model, we evaluated one-factor, bi-factor, four-factor and five factor models.

The validity of the proposed three-factor structure was then examined on the second random subsample using CFA and the WLSMV estimator (theta parameterization). The goodness-of-fit was evaluated using the comparative fit index (CFI), the Tucker-Lewis index (TLI), the root-mean-square approximation error (RMSEA) with its 90% confidence interval (CI), and the PClose. We used the following recommendations [55] to assess the model fit: TLI and CFI values ≥ 0.95 suggested excellent fit, TLI and CFI values ≥ 0.90 suggested acceptable fit; RMSEA values ≤ 0.06 suggested excellent fit, RMSEA values ≤ 0.08 suggested acceptable fit. The PClose is a measure of the p-value considering the null hypothesis that the RMSEA estimate is actually below 0.05. Hence, with a PClose ≥ 0.05 the null hypothesis that the RMSEA is actually below 0.05 is not rejectable, and the fit of the model is confirmed.

Construct validity was examined by computing Spearman correlation coefficients between the observed NPSS scores and the additional measures administered to the sample of participants. In accordance with Cohen's [56] recommendations, correlations between 0.70 and 0.89 were deemed to be very strong, those between 0.50 and 0.69 were deemed to be strong, those between 0.30 and 0.49 were deemed to be moderate, and correlations between 0.10 and 0.29 were deemed to be weak.

The scales were retested on a second sample of participants as an initial phase in determining reliability. As a retest coefficient, the Spearman correlation between the observed scores at times 1 and 2 (after three weeks) was computed. The stability of scores was assessed analyzing the intraclass correlation coefficient (ICC). Acceptable coefficients of stability were considered retest values higher than 0.70 (i.e., at least 50% of shared variance).

In particular, regarding ICC, we followed the conservative recommendations that have been provided by Portney and Watkins [57]: values between 0.5 and 0.75 are regarded as "poor to moderate", values between 0.75 to 0.9 are regarded as "good". Whenever available, we estimated and reported the 95% confidence intervals. The two-part Cronbach's α, the Spearman-Brown coefficient, and the Guttman's Lambda 4 coefficient were calculated [58,59] as additional reliability indicators after the split-half approach was used to examine the reliability of the NPSS.

Inter-rater reliability was evaluated using Cohen's K statistics in order to evaluate content validity [60]. Rater reliability is substantial since it describes the degree to which the data obtained in our study are accurate representations of the variables being examined. Two independent researchers (A.P. and M.M.) rated the NPSS items. To calculate the inter-rater consensus, Cohen's K statistics were computed. Following the criteria outlined by Fleiss, Levin, and Paik [61] and Cicchetti [62], it was determined if the value for K statistics is fair (between 0.4 and 0.59), good (between 0.60 and 0.74), or excellent (higher than 0.74). The coefficient in our study was found to be excellent (k = 0.84). A convenience sample of 10 students was used to assess the face validity of [63]. Participants were asked to provide a clearer phrasing for any questions they thought were confusing. The research team then debated the comments until an agreement was achieved and the tool's final version was developed.

3. Results

In order to carry out exploratory analyses, factorability measures were determined to be appropriate (determinant = 0.008; Bartlett's test of sphericity: degrees of freedom (df) = 406, $\chi 2 = 5642.214$, $p < 0.0001$; KMO test = 0.913). We chose to carry out an EFA on the first random subsample in order to find a factor structure that could meet the requirements of an approximate simple structure [48,64] and a CFA as a following step on the second random subsample because we had to investigate the most suitable measurement model for the Italian NPSS without the support of prior knowledge. Nevertheless, before doing these analyses, we first examined the whole dataset to find redundant items and those with low SMC. Items were deemed redundant if their intercorrelation was more than |.707|, or more than 50% of the shared variance. This threshold was not surpassed by any items. Items with SMCs less than 0.10 may be removed from the item pool since they are unlikely to make a meaningful contribution to the measurement model [48]. There was not a single item that demonstrated an SMC below this limit.

We used the scree-test [49], the PA [50], and the MAP correlation statistic [51] to conduct dimensionality studies on the first random subsample. Five observed eigenvalues were larger than the 95th percentile of the associated random eigenvalues, according to the PA, and the scree-plot line seemed to flatten down at the fifth factor, suggesting the extraction up to five factors, respectively (Fig. 1). Yet, the first element was where the MAP statistic fell the most short (0.0112, 0.0111, 0.0110, 0.0127, 0.0123, 0.0114). It was therefore clear that a maximum of three, four or five factors may be appropriate.

[Figure 1]

The fit of these models was then evaluated using ESEM [54] through WLSMV estimation, GEOMIN rotation,

and theta parameterization. Table S1 reports the outcomes of the ESEM models. The one-factor and bi-factor ESEM model had a poor fit (one-factor: df = 223, χ2 = 595.353, CFI = 0.731, TLI = 0.733, RMSEA = 0.082 [0.081; 0.087]; bi-factor: df = 217, χ2 = 581.281, CFI = 0.865, TLI = 0.867, RMSEA = 0.055 [0.059; 0.057]). The four-factor and the five-factor ESEM model showed a somewhat greater fit (four-factor: df = 200, χ2 = 331.428, CFI = 0.958, TLI = 0.947, RMSEA = 0.032 [0.029; 0.035]; five-factor: df = 181, χ2 = 250.335, CFI = 0.978, TLI = 0.971, RMSEA = 0.025 [0.021; 0.029]), however, no item had a single loading in the first factor with a confidence interval fully over 0.32. The three-factor solution had an appropriate fit (df = 211, χ2 = 387.521, CFI = 0.945, TLI = 0.934, RMSEA = 0.035 [0.032; 0.039]) and was found to represent the best measurement model because we could identify at least six items per factor that had a single loading with a confidence interval fully over 0.32. The 29 items loaded on a three-factor model (Table 1).
[Table 1]
The validity of the proposed three-factor, bi-factor or four-factor structures was then examined on the second random subsample using CFA and the WLSMV estimator (Table 2). The findings showed a poor fit for the bi-factor (df = 184, χ2 = 600.137, CFI = 0.921, TLI = 0.914, RMSEA = 0.082 [0.077; 0.087], PCLOSE < 0.001) and the four-factor model (df = 181, χ2 = 422.493, CFI = 0.954, TLI = 0.950, RMSEA = 0.063 [0.057; 0.068], PCLOSE < 0.001). The three-factor model confirmed an appropriate fit (df = 182, χ2 = 266.957, CFI = 0.984, TLI = 0.983, RMSEA = 0.037 [0.030; 0.044], PCLOSE = 0.874) (Fig. 2) and factor loadings (Table S2).
[Table 2]
[Figure 2]
The associations between the NPSS scores and the other scales in this research are shown in Table 3. The Italian version of the NPSS (Table S3) revealed very similar, positive moderate correlations with the SCS-SF and the USKS, indicating that higher scores on self-compassion and unconditional self-kindness are associated with a higher tendency to perceive a psychological neuroception of safety, supporting the convergent construct validity of the scale. The NPSS scores, on the other hand, were found to have a negative weak correlation with the BOA, SUP and BOA/SUB subscales of the BPQ-22 and with the STO questionnaire, indicating that higher scores on the neuroception of psychological safety are linked to a lower propensity to consciously perceive negative bodily feelings and to report traumatic experiences. Overall, these findings support the convergent and discriminant validity of the NPSS.
[Table 3]
Using a different participant sample, we next examined the scale's test-retest reliability. According to Table 4, which summarizes the findings, the test-retest correlation was 0.78, indicating that the scores were reasonably consistent across a three-week period. The intraclass correlation coefficient (ICC) test was also performed, and the results confirmed that the scores were consistent over a three-week period (ICC = 0.922). Additionally, we used the split-half method to evaluate reliability and found that the two split-half Cronbach's α were acceptable (split-half 1's = 0.913; split-half 2's = 0.868), along with the Spearman-Brown coefficient (CSB = 0.71) and the Guttman's Lambda 4 coefficient (GL4 = 0.72).
[Table 4]
4. Discussion
The current study's objective was to carry out a validation of the Italian version of the NPSS [19], evaluating its factor structure, content, convergent, discriminant and face validity, and reliability over time. Our findings showed that content, convergent, discriminant and face validity, as well as reliability over time were supported. Consistent with the original validation by Morton et al. [19], our data suggested a three-factor structure. The inflection point on the scree-plot advised that three factors may be extracted; however, in ESEM we also tested one-factor, bi-factor, four-factor and five-factor solution. Regarding newly generated items, saturation per item is recommended to be > 0.5, whereas for existing items, saturation for each item is recommended to be ≥ 0.6 [65]. Our findings meet these requirements since all factor loadings are greater than 0.61. According to the criteria established by Marsh et al. [55], the CFA results revealed an adequate fit for the three-factor model.
In order to analyze convergent validity, we found similar moderate positive Spearman correlations between the NPSS and the SCS-SF as well as between the NPSS and the USKS. Wouters-Soomers et al. [66] examined what is required in order to develop psychological safety at the individual level. Their findings demonstrated that for people to develop the meaningful relationships that foster psychological safety, they either need to have their fundamental needs met or show self-compassion. In addition, in order to measure, and enhance, psychological safety in mental health services, team-level surveys are administered that comprises proposed indicators of psychological safety as perceived compassion, as well as perceived institutional and managerial help [67]. Finally, it has been shown that among the key-points for ensuring a psychologically safe learning environment in a clinical context kindness represents a fundamental component [68]. Taken together, it may be hypothesized that these findings indicate that in order to develop, and maintain, psychological safety both self-compassion and self-kindness may be needed. Interestingly, it has been shown that loving-kindness may imply intentionally cultivating happiness and may result in a direct emotional experience that depends on the activation of the neurophysiological dopaminergic brain's reward system [69]. Analogously, it has been hypothesized that compassion stimulates the brain's positive emotion systems [70]. Overall, a focus on the activation of the positive affect system may be needed in order to foster and cultivate psychological safety.
Regarding discriminant validity, consistent with the previous findings, a weak negative correlation was revealed between NPSS and the STO questionnaire, highlighting the fact that when traumatic feelings are

acutely active in the body feelings of psychological safety are difficult to be elicited. Regarding Spearman correlations between NPSS and BPQ-22 subscales, we found negative weak correlations among NPSS and the three subscales of the BPQ-22, BOA, SUP and BOA/SUB. At first glance, it may seem a surprising result, however, inspecting the items of the BPQ-22, the wording of the items is biased towards a representation of unpleasant bodily feelings. Hence, in order to be able to feel psychological safety it may be needed that feelings of unpleasant bodily sensations are at low levels. In accordance with this, it has been shown that those who have a healthy body image and higher levels of body trusting also show higher levels of psychological safety and would be more resilient and able to handle stress and adversity, which will lead to sustained work performance [71]. In addition, in the context of interpersonal interactions, body trusting may improve work performance. In a community, pleasant behavior is encouraged and enhanced communication is made possible by psychological safety that results from body trusting [71]. Interestingly, mindfulness and self-reassurance have been shown to be able to reduce the neural activity of the negative affect system [70,72], and, therefore, they may be useful at reducing bodily traumatic feelings. Taken together, in order to develop, and maintain, a sense of psychological safety self-compassion and self-kindness may promote the activity positive affect system and, synergistically, self-reassurance and mindfulness, ma foster the reduction of the negative affect system and protect the individual from bodily traumatic feelings.

We assessed the test-retest reliability of the NPSS using data from a different sample of individuals who provided their answers three weeks later. Over a 3-week period, the NPSS scores were constant; in fact, the Spearman test-retest correlation was above 0.70, and the ICC was revealed to be above 0.80 in accordance with the conservative standards proposed by Portney and Watkins [57]. These findings suggested that the NPSS had a good test-retest reliability. In addition, utilizing a split-half approach, it was revealed that the two split-half Cronbach's alpha, the Spearman-Brown’s coefficient, and the Guttman's Lambda 4 coefficient were all above 0.71, supporting the NPSS's good test-retest reliability.

5. Conclusions

The following limitations should be taken into consideration when interpreting the results of this study: a) the scale's psychometric properties were first examined in a relatively large non-clinical sample obtained from the general Italian population; further studies are required to confirm the scale's three-factor structure and adequate reliability and validity in clinical samples; b) the participants' demographical characteristics were not representative of the general population, which may limit the generalizability of the results; c) three of the measures used in the research do not yet have an Italian published validation, as the papers are either being prepared for publication or are still in the publishing process; and d) criterion and nomological validity were not assessed, which should be evaluated in subsequent studies.

This study provided preliminary evidence that the NPSS is a valid and reliable three-dimensional scale to measure the neuroception of psychological safety. However, additional research is needed to confirm these results and validate the tool in languages different from Italian prior to its confident use in clinical and research contexts where this construct is of relevance.
