# Supplementary material for: Validation of the Italian version of the Neuroception of Psychological Safety Scale (NPSS)
Source: Heliyon. 2024 Mar 16;10(6):e27625. doi: 10.1016/j.heliyon.2024.e27625 (PMC10963227; doi:10.1016/j.heliyon.2024.e27625)
Supplement: Multimedia component 2 [file mmc2.docx]

# **Supplementary Materials**

*Table S1.*

*Goodness-of-fit indices for the Exploratory Structural Equation Modeling (ESEM) on the first random subsample (n = 173).*

| Model | χ^2^ | df | CFI | TLI | RMSEA [90% CI] |
| --- | --- | --- | --- | --- | --- |
| One-factor | 595.353 | 223 | .731 | .733 | .082 [.081; .087] |
| Bi-factor | 581.281 | 217 | .865 | .867 | .055 [.059; .057] |
| Three-factor | 387.521 | 211 | .945 | .934 | .035 [.032; .0.39] |
| Four-factor | 331.428 | 200 | .958 | 947 | .032 [.029; .0.35] |
| Five-factor | 250.335 | 181 | .978 | .971 | .025 [.021; .0.29] |

Note: all chi-square tests were significant at *p* < .001; df = degrees of freedom; CFI = Comparative Fit Index; TLI = Tucker Lewis Index; RMSEA = Root Mean Square Error of Approximation; CI = confidence interval.

*Table S2.*

*Loading matrix and factor correlations of the one-factor Confirmatory Factor Analysis model on the second random subsample (n = 165).*

|  | Item |  |
| --- | --- | --- |
| Factor 1 | NPSS01 | 0.70 [0.53; 0.61] |
|  | NPSS02 | 0.61 [0.47; 0.55] |
|  | NPSS03 | 0.70 [0.55; 0.63] |
|  | NPSS04 | 0.80 [0.68; 0.77] |
|  | NPSS05 | 0.75 [0.60; 0.69] |
|  | NPSS06 | 0.68 [0.56; 0.64] |
|  | NPSS07 | 0.53 [0.54; 0.64] |
|  | NPSS08 | 0.62 [0.50; 0.58] |
|  | NPSS09 | 0.74 [0.70; 0.80] |
|  | NPSS10 | 0.71 [0.61; 0.70] |
|  | NPSS11 | 0.73 [0.68; 0.78] |
|  | NPSS12 | 0.72 [0.71; 0.81] |
|  | NPSS13 | 0.63 [0.66; 0.76] |
|  | NPSS14 | 0.35 [0.34; 0.44] |
| Factor 2 | NPSS15 | 0.72 [0.50; 0.61] |
|  | NPSS16 | 0.76 [0.46; 0.54] |
|  | NPSS17 | 0.46 [0.35; 0.45] |
|  | NPSS18 | 0.62 [0.55; 0.68] |
|  | NPSS19 | 0.57 [0.42; 0.52] |
|  | NPSS20 | 0.72 [0.60; 0.72] |
|  | NPSS21 | 0.72 [0.59; 0.71] |
| Factor 3 | NPSS22 | 0.72 [0.71; 0.82] |
|  | NPSS23 | 0.70 [0.58; 0.67] |
|  | NPSS24 | 0.76 [0.60; 0.69] |
|  | NPSS25 | 0.78 [0.75; 0.85] |
|  | NPSS26 | 0.72 [0.71; 0.82] |
|  | NPSS27 | 0.79 [0.70; 0.80] |
|  | NPSS28 | 0.69 [0.58; 0.67] |
|  | NPSS29 | 0.77 [0.72; 0.83] |

Note: Bracketed values and the 95% confidence interval of the loading estimate.
