# Supplementary material for: Validation of the Italian version of the Neuroception of Psychological Safety Scale (NPSS)
Source: Heliyon. 2024 Mar 16;10(6):e27625. doi: 10.1016/j.heliyon.2024.e27625 (PMC10963227; doi:10.1016/j.heliyon.2024.e27625)
Supplement: Multimedia component 3 [file mmc3.docx]

*Table S3.*

*Italian version of the Neuroception of Psychological Safety Scale (NPSS).*

**Neuroception of Psychological Safety Scale (NPSS)**

Per favore, valuti quanto le seguenti affermazioni descrivano in maniera appropriata i suoi sentimenti vissuti durante la scorsa settimana.

Fortemente in disaccordo (punteggio = 1), In disaccordo (punteggio = 2), Né d'accordo o in disaccordo (punteggio = 3), D'accordo (punteggio = 4), Completamente d'accordo (punteggio = 5).

|  |  | Punteggio |
| --- | --- | --- |
| 1 | Mi sono sentito apprezzato |  |
| 2 | Mi sono sentito a mio agio nell'esprimermi |  |
| 3 | Mi sono sentito accettato dagli altri |  |
| 4 | Mi sono sentito compreso |  |
| 5 | Sentivo che gli altri mi capissero |  |
| 6 | Mi sono sentito rispettato |  |
| 7 | Vi era qualcuno che mi faceva sentire al sicuro |  |
| 8 | Vi era qualcuno di cui potevo fidarmi |  |
| 9 | Mi sono sentito confortato dagli altri |  |
| 10 | Mi sono sentito ascoltato dagli altri |  |
| 11 | Sentivo che le persone avrebbero fatto del loro meglio per aiutarmi |  |
| 12 | Sentivo che gli altri si prendessero cura di me |  |
| 13 | Mi sono sentito desiderato |  |
| 14 | Non mi sono sentito giudicato dagli altri |  |
| 15 | Mi sono sentito in grado di empatizzare con le altre persone |  |
| 16 | Mi sono sentito in grado di confortare un'altra persona se necessario |  |
| 17 | Ho sentito compassione per gli altri |  |
| 18 | Ho desiderato aiutare gli altri a rilassarsi |  |
| 19 | Ho sentito come se fossi in grado di poter confortare una persona amata |  |
| 20 | Mi sono sentito così connesso agli altri che ho desiderato aiutarli |  |
| 21 | Sentivo di volermi prendere cura degli altri |  |
| 22 | Sentivo che il mio battito cardiaco fosse stabile |  |
| 23 | Riuscivo a respirare senza sforzo |  |
| 24 | Sentivo che la mia voce fosse normale |  |
| 25 | Ho sentito il mio corpo rilassato |  |
| 26 | Sentivo che il mio stomaco fosse calmo |  |
| 27 | La mia respirazione era stabile |  |
| 28 | Mi sono sentito in grado di rimanere fermo |  |
| 29 | Ho sentito il mio volto rilassato |  |

**Scoring**:

NPSS total scoring

Sum of item scoring: item 1 to 29

Max total score: 145

NPSS subscale scoring

*Social Engagement* subscale: item 1 to 14

Max subscale score: 70

*Compassion* subscale: item 15 to 21

Max subscale score: 35

*Bodily sensations* subscale: item 22 to 29

Max subscale score: 40
